# Sample pooling for real-time PCR detection and virulence determination of the footrot pathogen *Dichelobacter nodosus*

**DOI:** 10.1007/s11259-017-9686-9

**Published:** 2017-03-25

**Authors:** Sara Frosth, Ulrika König, Ann-Kristin Nyman, Anna Aspán

**Affiliations:** 1 Department of Biomedical Sciences and Veterinary Public Health, Swedish University of Agricultural Sciences, P. O. Box 7036, SE-750 07 Uppsala, Sweden; 2 Department of Microbiology, National Veterinary Institute (SVA), SE-751 89 Uppsala, Sweden; 3 Farm and Animal Health, Kungsängens gård, SE-753 23 Uppsala, Sweden; 4 Department of Animal Health and Antimicrobial Strategies, National Veterinary Institute (SVA), SE-751 89 Uppsala, Sweden

**Keywords:** *Dichelobacter nodosus*, Ovine footrot, Pooling of samples, Real-time PCR, Virulence, *aprV2/B2*

## Abstract

*Dichelobacter nodosus* is the principal cause of ovine footrot and strain virulence is an important factor in disease severity. Therefore, detection and virulence determination of *D. nodosus* is important for proper diagnosis of the disease. Today this is possible by real-time PCR analysis. Analysis of large numbers of samples is costly and laborious; therefore, pooling of individual samples is common in surveillance programs. However, pooling can reduce the sensitivity of the method. The aim of this study was to develop a pooling method for real-time PCR analysis that would allow sensitive detection and simultaneous virulence determination of *D. nodosus*. A total of 225 sheep from 17 flocks were sampled using ESwabs within the Swedish Footrot Control Program in 2014. Samples were first analysed individually and then in pools of five by real-time PCR assays targeting the *16S rRNA* and *aprV2/B2* genes of *D. nodosus*. Each pool consisted of four negative and one positive *D. nodosus* samples with varying amounts of the bacterium. In the individual analysis, 61 (27.1%) samples were positive in the *16S rRNA* and the *aprV2/B2* PCR assays and 164 (72.9%) samples were negative. All samples positive in the *aprV2/B2* PCR-assay were of *aprB2* variant. The pooled analysis showed that all 41 pools were also positive for *D. nodosus 16S rRNA* and the *aprB2* variant. The diagnostic sensitivity for pooled and individual samples was therefore similar. Our method includes concentration of the bacteria before DNA-extraction. This may account for the maintenance of diagnostic sensitivity. Diagnostic sensitivity in the real-time PCR assays of the pooled samples were comparable to the sensitivity obtained for individually analysed samples. Even sub-clinical infections were able to be detected in the pooled PCR samples which is important for control of the disease. This method may therefore be implemented in footrot control programs where it can replace analysis of individual samples.

## Introduction

*Dichelobacter nodosus,* a fastidious Gram-negative bacterium, is the principal cause of ovine footrot (Beveridge 1941; Kennan et al. 2011). This is a transmissible disease that affects the epidermis of the feet and often results in lameness (Beveridge 1941). The disease has a worldwide occurrence in sheep and the economic impact on the sheep industry is substantial in terms of production losses and costs for treatment and prevention (Bennett and Hickford 2011; Green and George 2008; Marshall et al. 1991). Disease severity can range from inflammation of the interdigital skin (benign footrot) to complete underrunning of the hoof horn (virulent footrot) (Beveridge 1941; Egerton et al. 1969). The virulence of the infecting *D. nodosus* strain is an important factor for disease severity (Stewart et al. 1986). Two separate variants of the bacterium—virulent and benign—have recently been demonstrated by whole genome sequencing, and they have been shown to correlate with presence of *aprV2* and *aprB2*, respectively (Kennan et al. 2014). The presence of virulent and benign strains varies between countries; virulent strains are the most common variant in UK sheep (Maboni et al. 2016; Moore et al. 2005) while benign strains predominate in Swedish sheep (Frosth 2016). Inconsistencies between *D. nodosus* virulence and clinical signs have also been reported (Frosth et al. 2015; Moore et al. 2005; Stäuble et al. 2014b).

Detection and virulence determination of *D. nodosus* can today be achieved by real-time PCR analysis (Frosth et al. 2015; Frosth et al. 2012; Stäuble et al. 2014a). As analysis of a large number of samples may be required for an accurate diagnosis in large sheep flocks or to establish that a flock is free of footrot; this is both time consuming and costly. Therefore, pooling of individual samples is a common strategy of surveillance programs and is implemented, for example, in the Swedish Control Programs for *Salmonella* and *Campylobacter* (National Veterinary Institute 2016). However, pooling of samples can lead to a reduction in diagnostic sensitivity and should be evaluated before it is used. Hence, the aim of this study was to develop and evaluate a pooling method for real-time PCR analysis that would allow sensitive detection and simultaneous virulence determination of *D. nodosus*.

## Materials and methods

A total of 225 sheep from 17 flocks were sampled at routine inspections within the Swedish Footrot Control Program (Farm and Animal Health 2016) in September 2014. All feet were clinically examined and scored according to the footrot scoring system described by Stewart and Claxton (1993). Six flocks were considered affected by footrot and eleven flocks healthy or unaffected, according to the Swedish definition of footrot as a score ≥ 2 lesions. ESwabs (Copan Innovation Ltd., Brescia, Italy) were used to sample the interdigital skin of one foot per sheep. The swabs were sent to the National Veterinary Institute (SVA) by regular mail where they arrived within 1–3 days. There the swabs were shaken on a Thermomixer Comfort (Eppendorf, Hamburg, Germany) for 5 min at 700–800 rpm to evenly distribute bacteria in the transport medium; samples were then divided into two aliquots of 400 μl each. One aliquot was subjected to DNA-extraction on the EZ1 Advanced (Qiagen, Hilden, Germany) after a pretreatment step that included centrifugation (Frosth et al. 2012). In short, the bacteria were pelleted by centrifugation for 5 min at 13000 g and lysed by G2-buffer (Qiagen), proteinase K (Qiagen) and heat before automated DNA-extraction. The extracted DNA was used for detection and virulence determination of *D. nodosus (16S rRNA* and *aprV2/B2* genes, respectively) by real-time PCR assays as described in Frosth et al. (2015). Amplification was performed in an Applied Biosystems 7500 Fast Real-Time PCR System (Thermo Fisher Scientific Inc., Waltham, MA, USA) and the results were analysed with the 7500 software v2.0.6 using a manually set threshold of 0.1. Samples were defined as positive if they generated probe-specific fluorescent signals of quantification cycle (Cq) <40. This reflects the GLP in the diagnostic laboratory at SVA, were Cq-values between 38 and 40 are regarded as indicative of a possible presence of the bacterium, and the clinical veterinarian is in this case advised to do repeated sampling in the flock to confirm or rule out the diagnosis. Thus, loss of sensitivity even at these high Cq-values is undesirable, and was therefore included in the evaluation of the performance of the pooling method. Presence of either *aprV2* (virulent) or *aprB2* (benign) genes was counted as a positive outcome in the virulence PCR.

After determining which individual samples were positive or negative for *D. nodosus,* the other 400-μl aliquots from each sample were pooled in groups of five, comprising four negative and one positive *D. nodosus* samples. Based on the Cq-values obtained in the *aprV2/B2* PCR-assays for each individual sample, the pooled samples were divided into three categories; high, intermediate or low amounts of *D. nodosus* added to the pool (high = Cq <25 *n* = 10; intermediate = Cq 25–29.9 *n* = 21; and low = Cq ≥30 *n* = 10). Either of the two PCR-assays, *aprV2/B2* or *16S rRNA*, could have been used for estimation of the amount (high, intermediate or low) of *D. nodosus* in the samples. The *aprV2/B2* assay was chosen since it is the slightly less sensitive of the two, possibly due to a lower copy number in the genome; if it worked this would ensure that the pooling would work well for either assay. The pooled samples were centrifuged and subjected to DNA-extraction and PCR amplification as for the individual analysis. The only difference from the individual sample handling was that the starting volume of the transport medium was 2 ml instead of 400 μl. The individual analysis of each sample was performed on the same day that the samples arrived at the laboratory whereas the aliquots intended for pooling were stored at 4 °C until used. The majority of the positive *D. nodosus* aliquots were pooled and analysed on the same day as the corresponding individual analysis, but some of the aliquots and especially negative ones were, for practical reasons, stored for up to six days prior to the pooled analysis. The descriptive statistics (as box-and-whisker graphs) were made in Stata statistical software (StataCorp LP; Release 13.1; College Station, TX, USA).

## Results

**Fig. 1:**
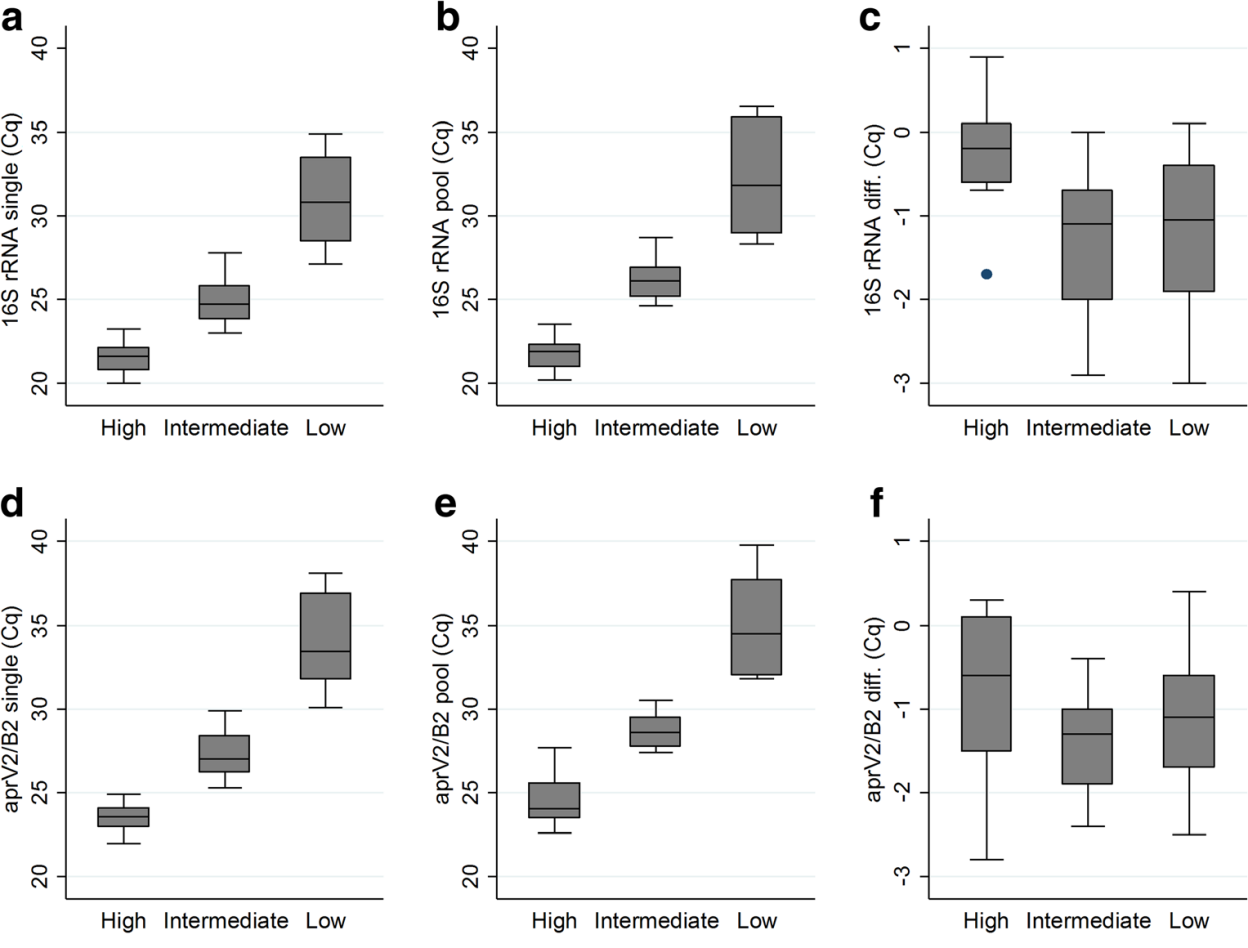
Box-and-whisker plots of the quantification cycle (Cq) values for individual (*n* = 41, Figs. a and d) and pooled samples (pools of five, the same positive samples as in the individual analysis but combined with four negative samples, Figs. b and e). Samples were analysed for *16S rRNA* (Figs. a, b and c) and *aprV2/B2* (Figs. d, e and f) by real-time PCR assays to detect *D. nodosus* and to determine virulence*,* respectively. Figs. c and f show the difference in Cq-values for the individual and pooled analysis. High, intermediate and low indicates the amount of *D. nodosus* bacteria in the samples as estimated by the *aprV2/B2* PCR-assay (high Cq <25 *n* = 10; intermediate Cq 25–29.9 *n* = 21; and low Cq ≥30 *n* = 10). The box shows the lower, median and upper quartiles, and the whisker shows the upper and lower adjacent values (1.5 inter-quartile range from the lower and upper quartiles, respectively). The dots are outliers

In the individual analysis, 61 (of 225, 27.1%) samples were positive in the *16S rRNA* and the *aprV2/B2* PCR assays and 164 (of 225, 72.9%) samples were negative in both. All samples positive in the *aprV2/B2* PCR-assay in this study were of *aprB2* variant (benign). Quantification cycle values for the 41 *D. nodosus* positive samples that were later pooled with negative samples can be seen in Figs. 1a and d. On flock level, *D. nodosus* was detected in 8 (47.1%) of the 17 flocks. Of these 8 flocks, 6 had clinical signs of footrot whereas 2 flocks had been assessed as healthy or unaffected.

In the pooled analysis, where a single *D. nodosus* positive sample was combined with four negative ones, all 41 were positive in both real-time PCR assays (Figs. 1b and e). A comparison of the Cq-values from the individual and pooled analysis for each sample showed an average difference of −1.1 Cq-value for the *16S rRNA* gene and −1.2 for the *aprB2* gene (Fig. 1c and f).

## Discussion

Pooling of swab samples for real-time PCR can provide cost-effective yet sensitive detection and virulence determination of *D. nodosus.* The pooling method developed in this study can be used as a diagnostic tool in footrot control programs and can be particularly useful in sheep flocks with unclear clinical signs. Eleven of the 17 flocks in our study were judged as healthy, but our method was able to detect *D. nodosus* in 2 of them. These two flocks have been inspected twice since sampling, after one and two years respectively, and they have not developed any clinical signs. The method is currently being used within the Swedish Footrot Control Program in all flocks with clinical signs of footrot and in all newly enrolled sheep flocks regardless of clinical signs due to the results of this study (National Veterinary Institute 2016).

Pooling of individual samples is commonly used to monitor diseases at flock level (National Veterinary Institute 2016). In spite of this, there are few publications on the subject, although it is important that methods used in practice should be thoroughly evaluated. A disadvantage with pooling is that a positive sample can be diluted if mixed with samples negative for the disease-causing agent, leading to a reduction in diagnostic sensitivity. In this study, the diagnostic sensitivity of the pooled and individual samples was the same, which was probably due to the concentration step that we implemented prior to bacterial lysis and DNA-extraction. The concentration step consisted of centrifugation and it was performed on both the individual and the pooled samples. A slight reduction of the analytical sensitivity for the pooled samples was seen as indicated by an increase of the Cq-values of the real-time PCR assays by approximately 1.1–1.2. However, it is unlikely that this is of any practical importance because the *D. nodosus* positive samples sent to the diagnostic laboratory at SVA, are seldom at the detection limit of the assays (Sara Åkerström, SVA, personal communication).

The use of only the *aprV2/B2* assay for both detection and virulence determination of *D. nodosus* has been proposed by Stäuble et al. (2014a) but neither their assay nor the very similar one in this study, contains an internal amplification control, meaning that there is a risk of false negative results. Hence a separate assay, targeting the *16S rRNA* gene, was used for detection of *D. nodosus* in this study (Frosth et al. 2015). Moreover, using the *16S rRNA* assay as a complement to the *aprV2/B2* assay means that isolates with possible mutations in the *aprV2/B2* genes will not be missed. The possibility of finding variants of *aprV2/B2* other than the two so far reported (Riffkin et al. 1995) is probably low, especially since *D. nodosus* is genetically highly conserved (Kennan et al. 2014). Nonetheless, running the two PCR-assays in parallel resulted in the discovery of a *D. nodosus* isolate with another amino acid in position 92 of AprV2/B2 than the described tyrosine and arginine (Frosth 2016). The *16S rRNA* and the *aprV2/B2* PCR-assays can be run simultaneously on the same assay plate since they both use the same PCR-program.

None of the swab samples in this study contained virulent *D. nodosus;* which is not surprising since very few virulent *D. nodosus* have so far been found in Sweden (Frosth 2016). However, there is every reason to believe that the assay works equally well for virulent *D. nodosus*. In a previous evaluation, the *aprV2* gene variant amplified and exhibited the same limit of detection as the *aprB2* gene variant (Frosth 2016; Frosth et al. 2015). Furthermore, the *aprV2/B2* PCR-assay has been successfully used on samples from UK sheep where the majority contained the *aprV2* variant (Maboni et al. 2016).

A new swab was used for each foot that was sampled and the pooling was conducted at the laboratory. This avoids possible spread of the disease within the flock and ensures consistency in the sampling procedure. However, separate swabs were not used for the individual and pooled analysis, since that would have meant repeated sampling of the same foot which might influence the results. This approach also provides the opportunity for an individual analysis to be performed after the pooled analysis, for identification of specific positive animals in the pool.

In conclusion, our pooling procedure (five individual swabs) had the same diagnostic sensitivity as individual swab samples in the real-time PCR assays for the detection and virulence determination of *D. nodosus.* Even sub-clinical infections were able to be detected in the pooled PCR samples which is important for control of the disease. The method is a potentially useful diagnostic tool in footrot control programs where it can replace analysis of individual samples.
